# Immune cell deconvolution of bulk DNA methylation data reveals an association with methylation class, key somatic alterations, and cell state in glial/glioneuronal tumors

**DOI:** 10.1186/s40478-021-01249-9

**Published:** 2021-09-08

**Authors:** Omkar Singh, Drew Pratt, Kenneth Aldape

**Affiliations:** 1 Laboratory of Pathology, Center for Cancer Research, National Cancer Institute, National Institutes of Health, Bethesda, MD 20892 USA; 2 Department of Pathology, University of Michigan, Ann Arbor, MI 48109 USA

**Keywords:** Deconvolution, Tumor microenvironment, Genomic aberrations, Immunotherapy

## Abstract

It is recognized that the tumor microenvironment (TME) plays a critical role in the biology of cancer. To better understand the role of immune cell components in CNS tumors, we applied a deconvolution approach to bulk DNA methylation array data in a large set of newly profiled samples (n = 741) as well as samples from external data sources (n = 3311) of methylation-defined glial and glioneuronal tumors. Using the cell-type proportion data as input, we used dimensionality reduction to visualize sample-wise patterns that emerge from the cell type proportion estimations. In IDH-wildtype glioblastomas (n = 2,072), we identified distinct tumor clusters based on immune cell proportion and demonstrated an association with oncogenic alterations such as *EGFR* amplification and *CDKN2A/B* homozygous deletion. We also investigated the immune cluster-specific distribution of four malignant cellular states (AC-like, OPC-like, MES-like and NPC-like) in the IDH-wildtype cohort. We identified two major immune-based subgroups of IDH-mutant gliomas, which largely aligned with 1p/19q co-deletion status. Non-codeleted gliomas showed distinct proportions of a key genomic aberration (CDKN2A/B loss) among immune cell-based groups. We also observed significant positive correlations between monocyte proportion and expression of PD-L1 and PD-L2 (R = 0.54 and 0.68, respectively). Overall, the findings highlight specific roles of the TME in biology and classification of CNS tumors, where specific immune cell admixtures correlate with tumor types and genomic alterations.

**Supplementary Information:**

The online version contains supplementary material available at 10.1186/s40478-021-01249-9.

## Introduction

Glial and glioneuronal tumors represent a wide range of tumor types with distinct biology and clinical outcomes and are currently assigned WHO grades 1–4 based on histopathologic and molecular features [1, 53]. The tumor microenvironment (TME) is a critical element in glioma biology and has been shown to alter sensitivity to immune-based therapies [17, 86]. In addition to immune cell burden, the specific cellular composition is also a critical feature of the TME and may influence key steps in tumor–immune interactions [39]. While the contribution of the TME to genomic instability in tumor cells has been previously investigated [20, 21, 71, 87], information is incomplete regarding the presence and/or activity of immunosuppressive or immunostimulatory cell types in gliomas, particularly in high-grade tumors [69]. Tumor infiltrating lymphocytes, or TILs, are often enriched in glioblastoma (GBM) with a mesenchymal gene expression signature and are strongly associated with mutations in *NF1* and *RB1* [75]. Conversely, TILs have been reported to be depleted in “classical” GBM (*EGFR*-amplified and PTEN-deleted) [75]. IDH-mutant gliomas often exhibit a less prominent TIL infiltration and decreased expression of the immune checkpoint molecule PD-L1 as compared to IDH wild types, reflecting a less immunosuppressive microenvironment [5]. Finally, recent evidence suggests tumor-associated macrophages (TAMs) may drive mesenchymal differentiation in GBM [76], consistent with reports that transcriptomic plasticity of GBM is significantly influenced by cellular heterogeneity in the tumor microenvironment [67, 78].

Aberrant DNA methylation is recognized as a key process for tumor development [42]. Distinct epigenetic profiles have been recognized in gliomas based on DNA methylation and, in combination with other somatic alterations, characterized clinically relevant subtypes: H3 K27, G34, IDH1, RTKI, RTKII, and mesenchymal [82]. An association with chromosomal alterations has been noted. For example, RTKI was found to be enriched for *PDGFRA* amplification and RTKII group showed combined chromosome 7 gain/chromosome 10 loss (+ 7/−10), *CDKN2A* loss and amplification of epidermal growth factor receptor (*EGFR*) [24, 81, 85]. IDH-wt subclass RTK III was predominantly reported in children and young adults, and correlates with pediatric high grade glioma, showing lower rates of TERT mutation and EGFR amplification as compared to adult-type GBMs [80]. Recently, DNA methylation profiling has emerged as a useful technique for tumor classification and the identification of novel subtypes in glial and glioneuronal tumors [10, 66].

Deconvolution of bulk tumors has typically involved in situ-based techniques, such as immunohistochemistry (IHC), as well as fluorescence-activated cell sorting (FACS), and more recently single-cell RNA sequencing (RNA-seq). Recent in silico techniques have allowed deconvolution on a much larger scale by utilizing high-throughput assays such as gene expression and DNA methylation microarrays [23, 33, 62, 63, 83, 84]. CIBERSORT is a widely-used reference-based deconvolution method used to de-convolve bulk transcriptomic data (e.g. RNA-seq) [37, 54, 95]. An adaptation of this method using DNA methylation data (MethylCIBERSORT) has been recently applied to pediatric CNS embryonal tumors and pediatric high-grade gliomas, revealing clinically and biologically relevant immune-based clusters in these select cohorts [12, 35]. Here, we sought to apply this deconvolution method to a large cohort of epigenetically defined, primarily adult glial and glioneuronal tumors with the aim of uncovering the relationship of tumor immune microenvironment with tumor subtype, canonical genetic alterations, and immune modulators.

## Materials and methods

### DNA methylation profiling

Samples (n = 741) were profiled as part of clinical methylation testing and analyzed as previously described by Capper et al. [10], and appropriate ethics approval was obtained for reporting. Briefly, bisulfite-converted genomic DNA was assayed on the Infinium MethylationEPIC kit (Illumina, USA), and the methylation profile was then classified using the Heidelberg methylation classifier. CNV profiles were derived from the R “conumee” package (http://bioconductor.org/packages/conumee/) as implemented in the classifier package. Batch effects were examined using variables of formalin-fixed, paraffin-embedded specimens (FFPE) versus frozen specimens. We also compared the 2 array types (450 k vs. EPIC). We did not observe major batch effects with respect to material type and array type.

### Data collection

We collated published literature to collect additional data for glial/glioneuronal tumor subtypes. The following publicly-available datasets (GSE, GEO database; E-MTAB, ArrayExpress) were included for downstream analyses: GSE104293 [4], GSE109381 [10], GSE124617 [6], GSE128654 [79], E-MTAB-5528 [57], GSE140124 [59], GSE61160 [60], GSE103659 [46], GSE111165 [7], GSE122994 [45], GSE131482, GSE157397 [25], GSE123678 [19], E-MTAB-5552 [58], GSE122920 [91], E-MTAB-3476 [2], GSE143843 [45], GSE137845 [34], GSE136361 [89], GSE73895 [41], GSE92462 [96], GSE60274 [22], E-MTAB-7802 [18], E-MTAB-5797 [28], GSE125450 [50], GSE119774 [56], E-MTAB-7490 [27], GSE135017 [36], E-MTAB-8390 [31], GSE152653 [55], GSE92579 [47], GSE104723, GSE116298 [90], E-GEOD-73895 and E-MTAB-7804 [18]. Our final dataset (N = 4052) also included samples from TCGA (N = 530) as well as samples processed within our laboratory (N = 741). Raw intensity data files (IDAT) for both 450 K and EPIC arrays were combined into a matrix with a common probe set (452,453 probes). All samples were processed and normalized by single sample noob function provided in the Minfi R package [3]. Sample annotation for tumor methylation class was performed using DKFZ classifier calibrated scores (v11b4) [10]. The final dataset (N = 4052) consisted of three broad glioma/glioneuronal tumor types: IDH-wt GBM (N = 2072), IDH-mut (N = 1178) and low-to-intermediate grade glioneuronal tumors (LIGGNT) (N = 802). LIGGNT is a broad category of glio-neuronal tumors consisting of 11 tumor subtypes: ANA-PA (N = 57), LGG-DIG/DIA (N = 13), LGG- DNT (N = 83), LGG-GG (N = 42), LGG-MYB (N = 56), LGG-PA/MID (N = 85), LGG-PA/PF (N = 216), LGG-PAGG/ST (N = 72), LGG-RGNT (N = 26), LGG-SEGA (N = 23), PXA (N = 129).

### Signature matrix development and validation

To construct the reference immune cell signature matrix, we collected methylation profile of pure non-neoplastic cell types. Raw data files (idat) were obtained from publicly-available sources, including B-cells (GSE110554,GSE49618, GSE35069, GSE88824) [9, 44, 72, 77], CD8 T-cells (GSE110554, GSE35069, GSE88824) [44, 72, 77], endothelial cells (GSE82234, GSE144804) [29, 73], monocyte, neutrophil, NK cells, eosinophils (GSE35069, GSE88824) [44, 72], glia (GSE66351) [32], neurons (GSE98203,GSE66351) [32, 48], CD4 T-cells and Treg (GSE49667) [94]. Microglia methylation profile was obtained from in-house profiling. Methylation profiles from 61 cancer cell lines were obtained from various sources (GSE128654, GSE68379, GSE152035, GSE134165; E-MTAB-9297, GSE122809, GSE137845, E-MTAB-9282), representing select types and subtypes of glial/glioneuronal tumors. Methylation data used to generate the signature matrix was processed and normalized by single sample noob function provided in the Minfi R package [3]. For immune cell deconvolution, we used the MethylCIBERSORT R package using the procedure as described by Chakravarthy et al. [12]. For feature selection, we used a modified function of FeatureSelect.V4 as described in Williamson et al. [35]. A maximum of top 100 features per pairwise comparison were selected with a median *β*-value difference of 0.2 and false discovery rate of 0.01. We selected 1,290 probes from the generated signature matrix differentiating 13 cell types: B-cells, cancer, CD4T, CD8T, endothelial, eosinophil, glia, microglia, monocyte, neuron, neutrophil, NK cells and Treg. To validate the matrix represents a homogeneous signature for each putative cell type, we performed dimensionality reduction (t-SNE) of pure reference cell types and heatmap with unsupervised clustering of selected probes and all cancer cell lines used to create the signature matrix. We generated scatter plots between cancer fraction and two purity measures (ESTIMATE purity and ABSOLUTE purity) [70] to demonstrate a correlation as a validation step for true cancer fraction estimation. Finally, we ran CIBERSORT on bulk tumor data using a Docker container with 1000 permutations without quantile normalization [63]. To validate our findings, we compared MethylCIBERSORT fraction output with gene expression based CIBERSORT proportion of six major cell types in data subset (N = 394) by using LM6 signature matrix [14]. We observed significant positive Pearson’s correlation (Additional file 1: Fig. 1) between similar cell type fractions [93].

### Dimensionality reduction and immune cell fraction estimation in tumor subtypes

We classified all samples (N = 4052) into three representative cohorts: IDH-wt (N = 2072), IDH-mut (N = 1178) and LIGGNT (N = 802). Our main objective was to investigate an immune cell fraction-based clustering pattern within these three major cohorts. We first investigated the clustering distribution by both including and excluding the inferred cancer proportion in each dataset (Additional file 2: Fig. 2). Prior to clustering we removed the cancer fraction and normalized the non-neoplastic cells (scaled from 0 to 1) and used immune cell fractions to generate UMAP for each cohort separately. The optimum numbers of clusters in each cohort was obtained using k-means clustering with Nbclust R package, providing 30 indices with which to determine the number of clusters [13]. Cluster selection process was based on the majority rule, which is available in the NbClust package. We compared three different methods; kmeans, ward.D and ward.D2 and finally selected kmeans clustering as a uniform approach to select optimum number of cluster (Additional file 3: Fig. 3). We next assessed overall immune cell proportions in each tumor subtype to determine methylation class-specific immune cell proportions (Additional file 4: Fig. 4a). We also investigated cancer proportion in each tumor subtype, and we found significant differences in cancer fraction of three major tumor cohorts (Additional file 4: Fig. 4b,c).

### Copy number variants

Somatic copy number variants were computed from raw signal intensities (IDAT) using the Conumee R package [40]. This method combines the intensity values of the methylated and unmethylated probes of each of the CpG sites and normalizes them to a set of control samples. CNV calling using DNA methylation array data has its own limitation such as it cannot rely on allele frequencies to define a copy-number neutral state baseline as described by Capper et al. [11] but still it is useful for large DNA methylation datasets. To investigate the presence of oncogenic gene amplifications and homozygous deletions events, we adopted the methodology described by Capper et al. [10]. We focused our analysis on select diagnostic and/or prognostic oncogenic events described in glial/glioneuronal tumors: *EGFR* amplification, *PTEN* deletion, *PDGFRA* amplification, *CDKN2A/B* deletion, 1p/19q codeletion, *MYCN* and *MDM2* amplification. Amplifications were defined as a log2ratio ≥ 1, and deletions defined as a log2ratio ≤ −0.8. We set these high cutoff values to identify true amplification/deletion events and to filter out small gain/loss. For 1p/19 codeletion, we considered co-deletion when both chromosome 1p and 19q showed log2ratio ≤ –0.20. MDM2 and CDK4 are frequently co-amplified genes in glioma, to validate our finding we examined all MDM2 amplified samples for CDK4 amplification. To investigate co-amplification we used average amplification cutoff (log2ratio ≥ 0.4) as described by Capper et  al. [11] and we observed co-amplification of MDM2 and CDK4 in more than 68% of samples.

### Immune cell proportion and PD-L1/2 gene expression

To investigate a possible association of monocyte proportion with gene expression and promoter methylation of the immune checkpoint ligands PD-L1 and PD-L2, we used a subset of the data (n = 594) which contained matched gene expression profiles (RNA-seq). RNA-seq trimmed mean M-values (TMM) was performed using the calcNormFactors function in the edgeR package [74]. Using the voom() function in limma, the counts were converted log2-counts-per-million (logCPM) and quantile normalized [49]. DNA methylation data was processed using single sample noob approach. We then selected promoter-associated probes in PD-L1/L2 and used the average beta value of all selected probes in these regions as the promoter methylation value.

### Survival analysis

Survival data was collected from original source of data (if available) as described in data collection sub-section of material and methods. To examine the association of immune cell proportion with overall survival, we performed Kaplan–Meier survival analyses with the log-rank test using the *survminer* and *survival* packages in R [43]. Samples with high proportion (> median) and low proportion (< median) were compared to see survival differences. Survival analysis was also performed to assess the cluster based survival differences in some cases (if survival data was available e.g. O-IDH).

## Results

### Identification and verification of methylation-derived immune cell populations

**Fig. 1:**
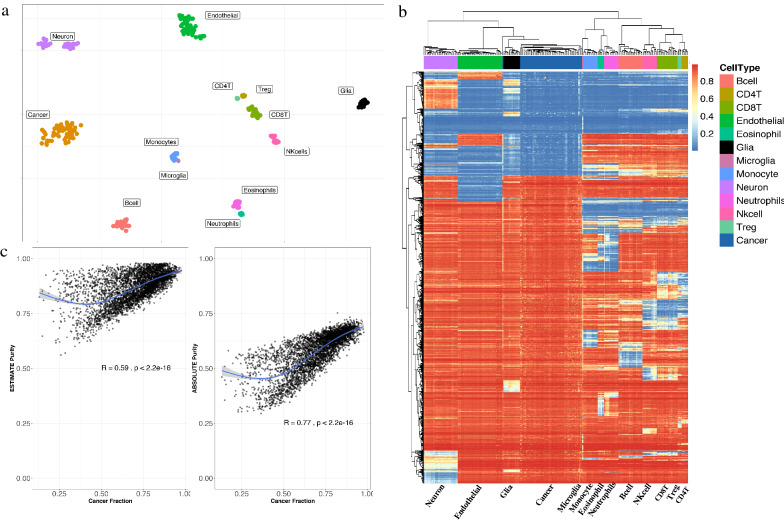
Signature matrix generation **a** t-SNE plot showing distinct clusters of methylation profiles of the pure reference cell types used to generate signature matrix. **b** Heatmap generated by all selected probes of signature matrix. **c** Scatterplot showing the estimated CIBERSORT cancer fraction (N = 4052) correlates significantly with purity (Estimate purity; R = 0.59; *p *value < 2.2 × 10–16, Absolute purity; R = 0.76; *p* value < 2.2 × 10–16)

A critical step in the deconvolution of bulk data is the appropriate choice of purified reference cell types and confirming a homogeneous population. Using MethylCIBERSORT, we constructed a signature matrix consisting of 1,290 probes distinguishing 13 specific cell types, as previously described: B-cells, CD4T, CD8T, Treg, NK cells, eosinophils, neutrophils, monocytes, microglia, endothelial cells, glial cells, neurons, and the cancer fraction. After generation of the signature matrix, we verified correct representation of cellular phenotypes by visualizing the profiles with t-SNE, which demonstrated an appropriate distinction between all purified reference groups (Fig. 1a). As confirmation, additional unsupervised hierarchical clustering of the signature matrix probes clearly distinguished these cell type clusters (Fig. 1b). We further investigated the association of the cancer fraction with two purity measures, ESTIMATE and ABSOLUTE (where available) and show a significant correlation with ESTIMATE-based (*R* = 0.59, *p* value < 2.2e−16) and ABSOLUTE purity (0.77, *p* value < 2.2e−16) (Fig. 1c).

### Immune cell deconvolution and clustering of IDH-wildtype diffuse gliomas

We first investigated the distribution of immune cell populations in a large set of IDH-wildtype diffuse glioblastoma (N = 2,072). This set consisted of seven methylation-defined glioma types: GBM-G34, GBM-MES, GBM-MID, GBM-MYCN, GBM-RTKI, GBM-RTKII and GBM-RTKIII that were defined using DKFZ DNA methylation classifier calibrated scores (version 11b4) [10]. From the deconvolution results, we calculated the overall mean proportion of immune cells (scaled from 0 to 1) in the IDH-wt dataset. The results indicated the average fraction of immune cells was proportionally highest for monocytes (31% of all non-cancer cells) and endothelial cells (11%) followed by CD8T (6%), B-cells (5%), neutrophils (5%), Treg (4%), microglia (3%), NK cells (2%), CD4T (1%) and eosinophils (1%). We also observed a significant contribution of non-immune cell such as glial cells (28%) and a minor contribution of neurons (1%).

**Fig. 2:**
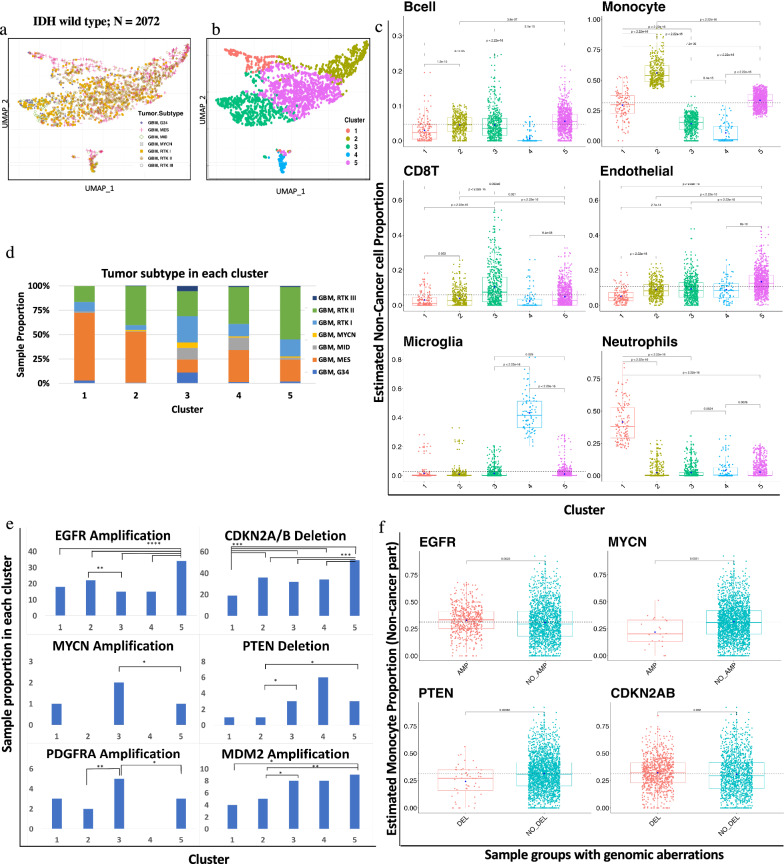
IDH-wt immune clustering showed five distinct clusters associated with immune cell proportions and key genomic aberrations. **a **UMAP clustering of seven tumor subtypes of IDH-wt tumor cohort (N = 2072) based on immune cell proportion (Non-cancer part scaled from 0 to 1) showed subtype specific clustering. **b** Five optimum number of cluster obtained by using k-means clustering with Nbclust method. **c** Six major Immune cell type proportions shown by boxplots for each cluster indicated significant difference between immune cells distribution across all cluster. Y axis represents non-cancer cell proportion of particular immune cell scaled from 0 to 1. Each box plots depicted, boxes indicate interquartile range with central bar indicating median and whiskers indicating the range. Blue dot represents mean value of the proportion. T-test and Wilcoxon test (*p* value shown) were used to calculate statistical significance. **d **Sample proportions of seven tumor subtypes in each cluster of IDH-wt tumor cohort (N = 2072). Distribution of tumor subtypes in each cluster calculated as percentage of samples represent specific tumor subtype. **e** Cluster specific genomic aberrations represented as proportion of samples undergoes for genomic changes. Horizontal bars between cluster bars with asterisks represent chi- square test based comparison with significant *p* value < (0.0001, 0.001, 0.01, 0.05, 1; symbols = "****", "***", "**", "*", "ns”). **f** Monocyte proportion shown by boxplots for various sample groups with genomic aberrations. T-test and Wilcoxon test (*p* value shown) were used to calculate statistical significance

We first examined clustering of IDH-wt GBM based on the calculated immune cell proportions. K-means consensus clustering was performed to identify an optimal number of clusters across all IDH-wt tumor samples (N = 2,072). The optimal number of clusters was determined by NbClust R package by providing 30 indices for determining the number of clusters [13]. Consensus clustering suggested five major clusters in the IDH-wt dataset (Fig. 2a,b). Cluster 1 was defined by a high proportion of neutrophils (median = 0.38), monocytes (median = 0.30), endothelial cells (median = 0.04) and B-cells (median = 0.02) (Fig. 2c). Interestingly, monocytes (median = 0.54) constituted the highest proportion in cluster 2 as compared to any other cluster, in addition to endothelial cells (median = 0.08) and B-cells (median = 0.04) (Fig. 2c). Cluster 3 demonstrated high levels of monocytes (median = 0.15), endothelial cells (median = 0.08), and CD8T (median = 0.07). Cluster 4 was defined by a high proportion of microglia (median = 0.41). Cluster 5 was similar to cluster 1 and was associated with high proportions of monocytes (median = 0.33), endothelial cells (median = 0.12), and B-cells (median = 0.05) (Fig. 2c). We observed that monocyte and endothelial proportions were dominant over any other cell type in the majority of clusters.

Immune cell proportions showed a distinct relationship with GBM methylation classes. As shown in Fig. 2d, the majority of samples in cluster 1 were GBM-MES (70%) and GBM-RTKII (16%). Conversely, cluster 5 showed an inverse pattern, with highest proportion of GBM-RTKII (54%) and a relatively low proportion of GBM-MES (22%). Representation of several of the less-common GBM subtypes, including GBM-G34, GBM-RTKII and GBM MYCN, were observed in cluster 3. A Sankey diagram demonstrating this relationship between DNA methylation class and immune-based clustering is shown in Additional file 5: Fig. 5.

### Immune clusters are associated with key oncogenic alterations in IDH-wt glioblastoma

Next, we investigated select copy number alterations in IDH-wt gliomas with respect to the defined immune clusters (Table. 1). We used *conumee* R package to analyze frequent genomic aberrations (amplifications/deletions) in GBM [40]. Cluster 5 showed a significantly higher proportion of samples harboring *EGFR* amplification and was approximately twice as frequent as tumors in clusters 3 and 4 (Fig. 2e). Tumors with homozygous deletion of *PTEN* were relatively enriched in cluster 4, and *MYCN* or *PDGFRA* amplification were enriched in cluster 3. To understand the significance and association of monocyte proportion in genomic aberrations we classified samples groups based on presence/absence of these alterations and compared the monocyte proportion between these groups (Fig. 2f). Interestingly, we found significant differences in monocyte proportions between sample groups (*EGFR*, *p* < 0.01; *MYCN*, *p* < 0.01, *PTEN*, *p* < 0.001, *CDKN2AB*, *p* < 0.05) based on genomic aberrations.

**Table 1:** Genomic aberrations, Immune cell proportion and tumor subtype in IDH-wt GBM cohort (N = 2072)

Cluster	Major cell Type	Genomic aberrations	Major Subtype
1	Neutrophil	Low CDKN2A/B deletion	GBM-MES
2	Monocyte	EGFR amplification	GBM MES GBM-RTKII
3	Monocyte CD8T	PDGFRA amplification* PTEN deletion MDM2 amplification MYCN amplification EGFR amplification	Mix RTK III
4	Microglia		Mix
5	Monocyte Endothelial B-cell	EGFR amplification* CDKN2A/B deletion* MDM2 amplification*	GBM-RTKII

^*^Most significant genomic changes as compared to other clusters

### Immune clusters are associated with glioma cell states in IDH-wt GBM

Previously, four main cellular states for IDH-wt GBM (AC-like, OPC-like, MES-like and NPC-like) have been identified based on single-cell RNA sequencing data [61]. Here, we used TCGA samples based on the availability of matched mRNA and methylation data and used CIBERSORTx to estimate the relative proportion of these cellular states. We observed significant differences in the proportion of cell states versus our immune cell-defined clusters. Clusters 3 and 5 were associated with a high proportion of OPC-like and AC-like malignant states, respectively (Additional file 6: Fig. 6), and Clusters 1 and 2 were populated by tumors with a high proportion of tumors with predominant MES-like cell state. We also analyzed tumor-specific survival probabilities in relation to immune cell proportion in tumor subtypes of IDH-wt cohort (Additional file 7: Fig. 7). We compared two groups based on median monocyte proportion in GBM-MES (N = 121) and GBM-RTKI (N = 52) and we observed poor overall survival in monocyte-high samples (*p* < 0.01 and *p* = 0.05, respectively) (Additional file 7: Fig. 7a, b). Similarly, we compared high/low endothelial proportion groups based on median proportion and found that samples with a high proportion of endothelial cells showed improved survival probability (*p* = 0.032) (Additional file 7: Fig. 7c) in GBM-MES.

### IDH-mutant diffuse gliomas

**Fig. 3:**
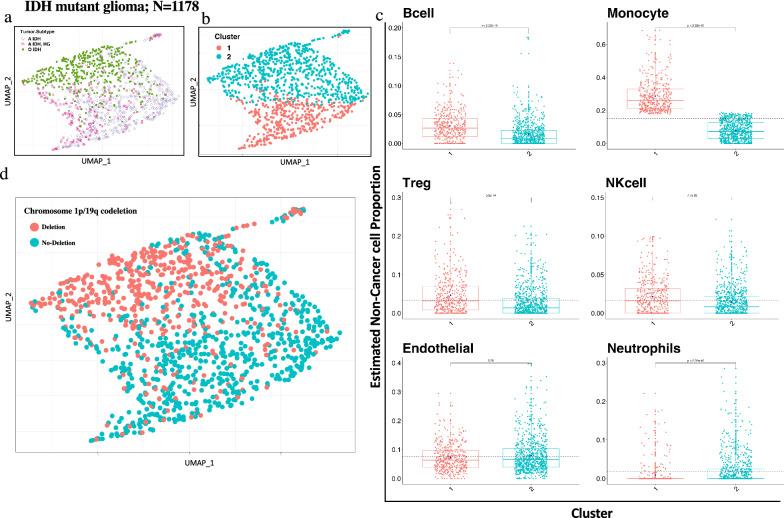
IDH-mutant glioma immune clustering showed two distinct clusters associated with 1p/19q status and different immune cell proportions. **a **UMAP clustering of three tumor subtypes of IDH-mut tumor cohort (N = 1178) based on immune cell proportion (Non-cancer part scaled from 0 to 1) showed subtype specific clustering. **b** Two optimum number of cluster obtained in IDH-mut cohort by using k-means clustering with Nbclust method. **c** Six major Immune cell type proportions shown by boxplots for each cluster indicated significant difference between immune cells distribution between two clusters. Y axis represents non-cancer cell proportion of particular immune cell scaled from 0 to 1. Each box plots depicted, boxes indicate interquartile range with central bar indicating median and whiskers indicating the range. Blue dot represents mean value of the proportion. T-test and Wilcoxon test (*p* value shown) were used to calculate statistical significance. **d** Copy number analysis of DNA methylation profile of IDH-mut cohort showed distinct distribution of samples with 1p/19q codeletion

The IDH-mutant cohort (N = 1178) was next examined and comprised the methylation classes O-IDH (oligodendroglioma), A-IDH (astrocytoma) and A-IDH-HG (high-grade astrocytoma). The presence of an IDH mutation was inferred from the methylation data. Surprisingly, visualization (UMAP) revealed apparent discrimination of these three methylation classes according to relative immune cell proportion (Fig. 3a). K-means consensus clustering revealed n = 2 optimal number of clusters across all IDH-mutant tumor samples (Fig. 3b), and corresponding differences in immune cell proportion were noted (Fig. 3c): cluster 1 was relatively overrepresented by monocytes and cluster 2 showing increased representation of endothelial cells. Overall, cluster 1 showed higher infiltration of immune cells, and as expected based on the methylation classifier results, was enriched in non-1p19q codeleted samples (Fig. 3d). Cluster 1 contained 62 co-deleted and 359 non-codeleted samples, as compared to cluster 2, which contained 437 co-deleted and 320 non-codeleted samples, revealing a significant difference in immune cell composition in these genomically-defined groups (p < 2.2e−16, chi-square test). Our results are in line with a recent study described association of IDH mutation and 1p19q co-deletion with tumor immune microenvironment [52]. Interestingly, we found that the overall immune cell proportions in IDH mutant tumors was lower than IDH wild type GBMs, although A-IDH-HG tumors had increased immune cell infiltration as compared to other IDH mutant gliomas (Additional file 4: Fig. 4a).

**Fig. 4:**
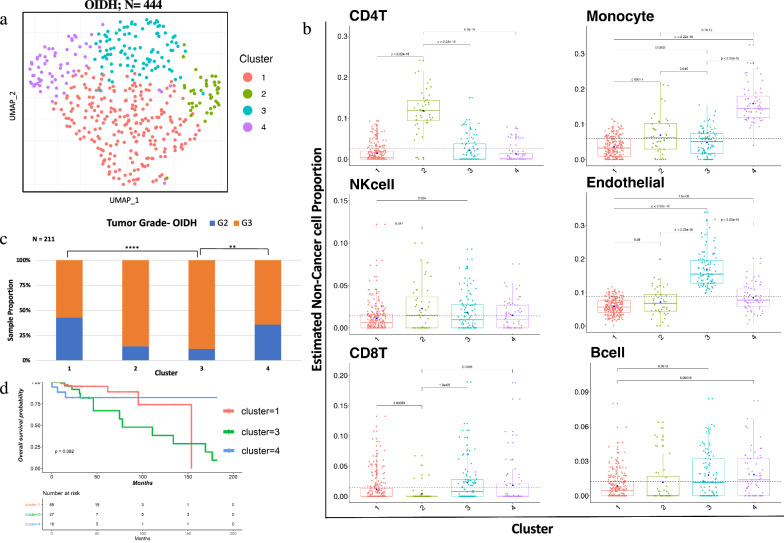
Immune clustering of IDH-mut oligodendroglioma (N = 444) showed four distinct clusters associated with tumor grade and patient outcome. **a **UMAP clustering of OIDH tumor cohort (N = 444) based on immune cell proportion (Non-cancer part scaled from 0 to 1) showed distinct clusters. **b** Six major Immune cell type proportions shown by boxplots for each cluster indicated significant difference between immune cells proportion across all cluster. Y axis represents non-cancer cell proportion of particular immune cell scaled from 0 to 1. Each box plots depicted, boxes indicate interquartile range with central bar indicating median and whiskers indicating the range. Blue dot represents mean value of the proportion. T-test and Wilcoxon test (*p* value shown) were used to calculate statistical significance. **c** Tumor grade distribution of samples across all clusters. Significance based on Fisher's exact test (cluster1-cluster3; *p* value = 0.000017, cluster3-cluster4; *p* value = 0.014). **d** Kaplan–Meier plot showing significant difference in overall survival between cluster 1 cluster 3 and 4

We next focused on oligodendroglioma samples in isolation (N = 444), where consensus clustering on immune cell proportion indicated four major clusters (Fig. 4a). Differences in individual cell types were noted (Fig. 4b). Cluster 2 and cluster 3 showed high proportion of grade 3 tumor samples as compared to cluster 1 and cluster 4, which consist of both grade 2 and grade 3 samples (Fig. 4c). Interestingly, significant survival differences were noted among immune-based groups (Fig. 4d).

**Fig. 5:**
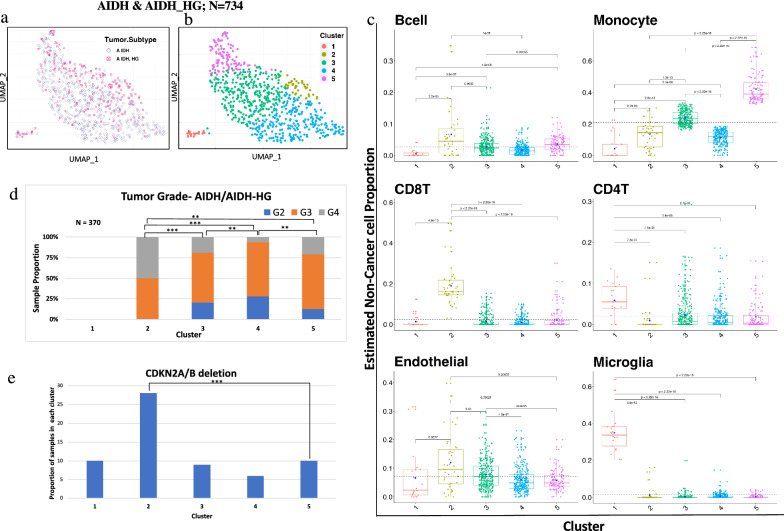
IDH-mut astrocytoma immune clustering showed five distinct clusters associated with tumor grade and CDKN2A/B deletion. **a** UMAP clustering of IDH-mutant astrocytoma cohort (N = 734) based on immune cell proportion (Non-cancer part scaled from 0 to 1) showed subtype specific clustering. **b** Five optimum number of cluster obtained in IDH-mut cohort by using k-means clustering with Nbclust method. **c** Six major Immune cell type proportions shown by boxplots for each cluster indicated significant difference between immune cells distribution across all cluster. Y axis represents non-cancer cell proportion of particular immune cell scaled from 0 to 1. Each box plots depicted, boxes indicate interquartile range with central bar indicating median and whiskers indicating the range. Blue dot represents mean value of the proportion. T-test and Wilcoxon test (*p* value shown) were used to calculate statistical significance. **d** Sample proportions as tumor grade 1, 2 & 3 in each cluster (N = 370; significance based on Fisher's exact test; *p* value < (0.0001, 0.001, 0.01, 0.05, 1; symbols = "****", "***", "**", "*", "ns”). **e** Cluster 2 and 5 showed significant difference between sample proportion with CDKN2A/B deletion. Horizontal bars between cluster bars with asterisks represent chi- square test based comparison with significant *p* value (< 0.001)

In IDH-mutant, non-1p/19q co-deleted gliomas (N = 734), we identified five optimal immune-based clusters (Fig. 5a, b). Significant differences were apparent for monocytes, CD8 T-cells, CD4 T-cells and microglia (Fig. 5c). When examining the distribution of histologic grade (Fig. 5d), there was a notable absence of low-grade (2) in cluster 2, defined by a high proportion of CD8 T-cells (Fig. 5c). As an additional analysis, we analyzed the proportion of tumors with homozygous loss of *CDKN2A/B* (a clinically relevant marker in these tumors), showing a higher proportion in cluster 2 consistent with the higher overall tumor grade in this cluster (Fig. 5e). Similarly, we also investigated heterozygous and homozygous deletion by selecting a range of log2ratio(homozygous < −0.8; heterozygous < −0.3) as separate event [26] and we found cluster 2 with higher number heterozygous/homozygous deletions as compared to other clusters (Additional file 8: Fig. 8). Overall, increased numbers of CD8 T-cells were associated with higher grade and *CDKN2A/B* homozygous deletion, highlighting the relationship of the TME with somatic alterations.

### Circumscribed and low-to-intermediate grade glial/glioneuronal tumors

**Fig. 6:**
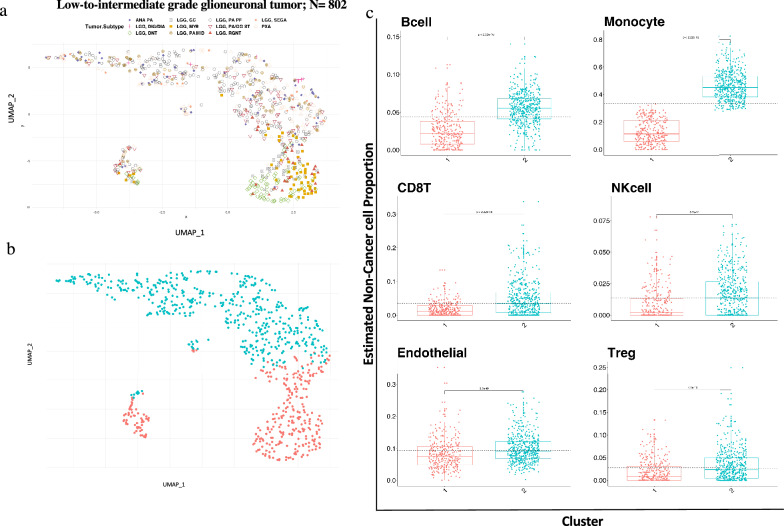
Immune clustering of Low-to-intermediate grade glioneuronal tumors (LIGGNT) showed distinct clusters associated with tumor type and different immune cell proportions. **a** UMAP clustering of eleven tumor subtypes of the LIGGNT cohort (N = 802) based on immune cell proportion (Non-cancer part scaled from 0 to 1) showed subtype specific clustering. **b** Two optimum number of cluster obtained in the LIGGNT cohort by using k-means clustering with Nbclust method. **c** Six major Immune cell type proportions shown by boxplots for each cluster indicated significant difference between immune cells distribution between two clusters. T-test and Wilcoxon test (*p* value shown) were used to calculate statistical significance

Immune-based clustering of low-to-intermediate grade glioneuronal tumors (LIGGNT) (N = 802) consisted of 11 DNA methylation-based types: anaplastic pilocytic astrocytoma/high grade astrocytoma with piloid features (ANA-PA), desmoplastic infantile ganglioglioma and astrocytoma (DIG-DIA), dysembryoplastic neuroepithelial tumor (DNT), ganglioglioma (GG), low-grade glioma with MYB/MYBL1 alteration (MYB), midline pilocytic astrocytoma (PA-MID), pilocytic astrocytoma, posterior fossa (PA-PF), supratentorial pilocytic astrocytoma/ganglioglioma (PA/GG-ST), rosette-forming glioneuronal tumor (RGNT), subependymal giant cell astrocytoma (SEGA), and pleomorphic xanthoastrocytoma (PXA) (Fig. 6a). K-means clustering and Nbclust showed an optimum number of two clusters in the LIGGNT cohort (Fig. 6b). There were distinct differences in the proportion of immune cells according to cluster group (Fig. 6c), including monocytes, neutrophils, and NKcells. Cluster 1 was enriched for MYB, DNT and GG samples, while cluster 2 was enriched for PA/PF, PXA, PA/MID and ANA-PA.

### Monocyte proportion is associated with PD-L1 and PD-L2 gene expression and promoter methylation in bulk methylation data

**Fig. 7:**
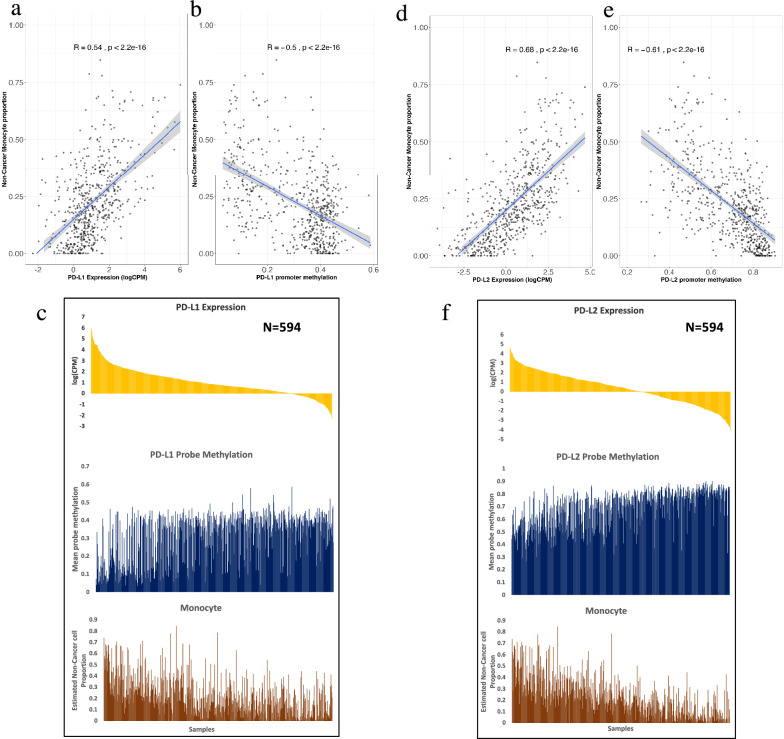
Association of monocyte proportion with PD-L1 and PD-L2 gene expression and gene promoter methylation (N = 594). **a** Scatterplot of Monocyte proportion against the gene expression of PD-L1 showed significant positive correlation. **b** Scatterplot of Monocyte proportion against the promoter methylation of PD-L1 showed significant negative correlation. **c** Bar plot of 594 sample arranged from high to low expression of PD-L1. Arranged samples showed probe methylation low to high (Opposite to gene expression) and monocyte proportion high to low, similar to gene expression. **d** Scatterplot of Monocyte proportion against the gene expression of PD-L2 showed significant positive correlation. **e** Scatterplot of Monocyte proportion against the promoter methylation of PD-L2 showed significant negative correlation as compared to PD-L1. **f** Bar plot of 594 sample arranged from high to low expression of PD-L2. Arranged samples showed probe methylation low to high (Opposite to gene expression) and monocyte proportion high to low, similar to gene expression

Consistent with prior studies [15, 35] we found that cells of the monocytic lineage were among the most abundant immune cell types in glial/glioneuronal tumors. Furthermore, the success of immune checkpoint inhibitors in lung carcinoma and melanoma [64, 65] has led to interest in characterizing the immunologic milieu in brain tumors. We therefore investigated the association between monocyte proportion and gene expression and promoter methylation of the immune checkpoints PD-L1 and PD-L2. Using a subset of samples (n = 594) with matched gene expression profiles, we observed a significant positive correlation between monocyte proportion and expression of PD-L1 and PD-L2 (R = 0.54; *p* < 2.2e−16 and 0.68; *p* < 2.2e−16, respectively). Consistently, we found high negative correlations (R = − 0.51 and − 0.61, respectively) between monocytes and promoter methylation of PD-L1 (Fig. 7a, b,c) and PD-L2 (Fig. 7d, e, f). After sorting samples in decreasing order of PD-L1 and PD-L2 expression, we found an expected trend of promoter methylation (low to high) and monocyte proportion (high to low) (Fig. 7).

## Discussion

Here we report results of a large-scale deconvolution analysis of > 4000 methylation-defined, primarily glial and glioneuronal tumors. We observed significant diversity in the tumor microenvironment composition among tumor subtypes and demonstrate an association with specific genomic aberrations, tumor grade and prognosis. Similar to prior studies, we found monocytes constitute one of the most abundant cell types in the immune microenvironment of gliomas [8, 16, 35, 68, 69, 88]. This was particularly evident in IDH-wt tumors, where an association with *EGFR* and *MDM2* amplification and CDKN2A/B deletion was observed in monocyte-rich clusters. Conversely, we observed a low monocyte proportion in *MYCN* amplified and *PTEN* deleted groups. The proportion of CD8 T-cells, another prominent immune cell type in high-grade gliomas, was associated with oncogene amplifications in *MYCN*, *PDGFRA*, and *MDM2*.

Studies of cell states in glioblastoma have previously demonstrated an association with chromosomal alterations [61]. We therefore investigated whether the immune-based clusters identified in the present study were associated with these cellular states. In the current study, we found the AC-like cell state was associated with immune clusters harboring *EGFR* amplification, while the OPC-like cell state was associated with *PDGFRA* amplification. The association of immune-based clusters with somatic alterations and cell states described by Suva et al. suggest a role of tumor microenvironment cellular constituents (monocytes, T/B-cells, endothelial cells) in contributing to these malignant cell states, suggesting cross-talk between tumor cell states and the immune microenvironment.

A recent study showed that IDH wild-type tumors associated with high degree of immune cell infiltration, and were suggested as immune-hot phenotype, whereas IDH mut-tumors with 1p/19q codeletion cohort showed low degree of immune cell infiltration as immune-cold phenotype [51]. Our Immune cell based clustering of IDH-mutant tumors also revealed low infiltration of immune cells in IDH-mut-codel associated with1p/19q codeletion.

Recently, it has been shown that CDKN2A homozygous deletion was associated with poorer outcome among IDH-mutant gliomas lacking 1p/19q codeletion (IDH-mutant astrocytoma) as well as among anaplastic oligodendrogliomas, IDH-mutant-1p/19q codeleted [3]. We also observed two major clusters with significant difference in CDKN2A/B deletion in IDH mutant astrocytoma cohort associated with high proportion of CD8 T, B-cell, and endothelial cells. We found in IDH-wt cohort that the cluster with high proportion of endothelial cells and B-cells prominently showed a greater number of samples with CDKN2A/B deletion and similarly in IDH-mut astrocytomas we found that in cluster 2 significant number of samples presented CDKN2A/B deletion.

Immune clustering of low-to-intermediate grade glioneuronal tumors (LIGGNT) (N = 802, 11 tumor subtypes) showed some promising outputs regarding tumor subtype specific overall infiltration of immune cells. We observed that several tumor subtypes, including MYB, DNT, GG and RGNT showed lower infiltration of immune cells as compared to other subtypes and these observations are in line with a recent study [35].

Given that cells of the monocyte lineage are one of the most dominant and key modulator of tumor microenvironment in glio-neuronal tumors, we examined the association of monocytes with programmed cell death ligands such as PD-L1 and PD-L2 gene expression, which plays crucial role in immunotherapy. It has been shown in literature that GBM EVs (extracellular vesicles) induce immunosuppressive monocytes, including myeloid-derived suppressor cells (MDSCs) and nonclassical monocytes (NCMs) [38] and glioblastoma stem cell-derived exosomes can induce M2 macrophages and PD-L1 expression on human monocytes [30]. Accordingly, it has been suggested that blocking PD-1/PD-L1 pathway could be potential new treatment study in glioma [92]. We investigated the possible role of monocyte proportion-relative gene expression and promoter methylation of the immune checkpoint PD-L1 and PD-L2 genes, we used a data subset (n = 594) sample with matched gene expression profiles. We observed significantly high positive correlations (R = 0.54 and 0.68, respectively) between monocyte proportion and expression of PD-L1 and PD-L2, in line with prior reports that monocytic cells can express these immune markers [38, 97]. Consistent with this, we found high negative correlations (R = − 0.51 and − 0.61, respectively) between monocytes and promoter methylation of PD-L1 and PD-L2 respectively. These results suggest a role of monocytic-lineage cells in immunotherapy response of gliomas, which warrants further investigation.

## Conclusion

In conclusion, our analysis relies on a large sample set of glial/glioneuronal tumors to demonstrate relationships of tumor immune microenvironmental factors with tumor type and key genomic aberrations. We also highlight the prominent role of monocytic lineage cells in these tumors, including associations with expression of immune checkpoint ligand PD-L1 and PD-L2 in these tumors. Results of this investigation provides insights for future investigation into glioma biology and immunotherapeutic approaches in gliomas.

## Supplementary Information

**Additional file 1**: To validate our MethylCIBERSORT output we selected a data subset (N=394) with available gene expression data. CIBERSORTx method was used with LM6 signature matrix to get fraction of six major cell types. We found significant positive correlations between MethylCIBERSORT fraction and CIBERSORTx derived fraction of six major cell types.

**Additional file 2**: Assessment of clustering pattern in all three major cohorts IDH-wt (N=2072), IDH-mutant (N=1178) and Low-to-intermediate grade glioneuronal tumors (LIGGNT) (N=802) by including and excluding cancer part and scaling non-cancer part from 0 to 1. (a) UMAP clustering for IDH-wt cohort with all cell type (including cancer). (b) UMAP clustering for IDH-wt cohort with all normal cell type scaled 0 to 1 (non-cancer part). (c) UMAP clustering for IDH-wt cohort with all immune cells type scaled 0 to 1 (non-cancer part). (d) UMAP clustering for IDH-mutant cohort with all cell type(including cancer). (e) UMAP clustering for IDH-mutant cohort with all normal cell type scaled 0 to 1 (non-cancer part). (f) UMAP clustering for IDH-mutant cohort with all immune cells type scaled 0 to 1 (non-cancer part). (g) UMAP clustering for LIGGNT cohort with all cell type (including cancer). (h) UMAP clustering for LIGGNT cohort with all normal cell type scaled from 0 to 1 (non-cancer part). (i) UMAP clustering for LIGGNT cohort with all immune cells type scaled 0 to 1 (non-cancer part).

**Additional file 3**: Cluster selection process was based on the majority rule, which is available in the NbClust package. We compared three different methods; kmeans, ward.D and ward.D2. (a) For each method we selected optimum number of clusters (given in brackets) proposed by maximum number of indices out of 30. We compared output of each method and found similar results. Finally, we selected kmeans clustering as a uniform approach to select optimum number of cluster (proposed by maximum number of indices out of 30) in each cohort. (b) In IDH wild type 11 indices proposed five cluster. (c) In IDH mutant type 8 indices proposed two cluster. (d) In O-IDH cohort, 6 indices proposed two and four clusters respectively. In this case we selected four cluster as ward.D2 method also suggested four clusters in O-IDH cohort. (e) In A-IDH/A-IDH-HG 8 indices proposed five clusters. (f) In LIGGNT 10 indices proposed two clusters.

**Additional file 4**: (a) Tumor subtype specific bar plot distribution of mean Immune cell proportion (Non-cancer part scaled 0 to 1, Glia and Neuron excluded). (b) Overall cell fractions of each cell type (Including Cancer, Glia and Neuron). (c) Cancer proportion shown by boxplots for each tumor subtype indicated significant differences. T-test and Wilcoxon test (*p* value shown) were used to calculate statistical significance.

**Additional file 5**: Sankey diagram-based associations between immune cells and tumor subtype in IDH-wt cohort (N=2072). Sankey plot showing proportions shared between each immune clusters and tumor subtype.

**Additional file 6**: Analysis of the IDH-wt glioblastoma cohort (N=100) shows cluster specific distribution of AC-like, OPC-like, MES-like and NPC-like cellular states, respectively. Tumor cell states were derived from single cell data of IDH-wild type GBMs (PMID: 31327527). A signature matrix was derived and applied to TCGA samples for which gene expression data were available. Cell state estimations for each sample were performed using CIBERSORTx. T-test and Wilcoxon test (p-value shown) were used to calculate statistical significance.

**Additional file 7**: Kaplan-Meier plot of (a) GBM-MES for Monocyte proportion high and low groups (High>median, Low<median). (b) GBM-RTK-I dataset with high and low proportion of monocytes (High>median, Low<median). (c) Kaplan-Meier plot for Endothelial cell high and low groups in GBM-MES (High>median, Low<median).

**Additional file 8**: To investigate homozygous and heterozygous deletion of CDKN2AB as a separate event we selected a range of log2ratio and annotated samples as an event of homozygous deletion, heterozygous deletion, and no deletion. Significance was calculated by applying Fisher's exact test; p-value <(0.0001, 0.001, 0.01, 0.05, 1; symbols ="****", "***", "**", "*", "ns”).

## Data Availability

The datasets presented in this study can be found in online repositories. The names of the repository/repositories and accession number(s) can be found in the Materials and Methods section. Newly profiled data is available from authors upon reasonable request.
