# Supplementary figures and images for: Immune cell deconvolution of bulk DNA methylation data reveals an association with methylation class, key somatic alterations, and cell state in glial/glioneuronal tumors
Source: Acta Neuropathol Commun. 2021 Sep 8;9:148. doi: 10.1186/s40478-021-01249-9 (PMC8425010; doi:10.1186/s40478-021-01249-9)

Supplementary Fig. 1

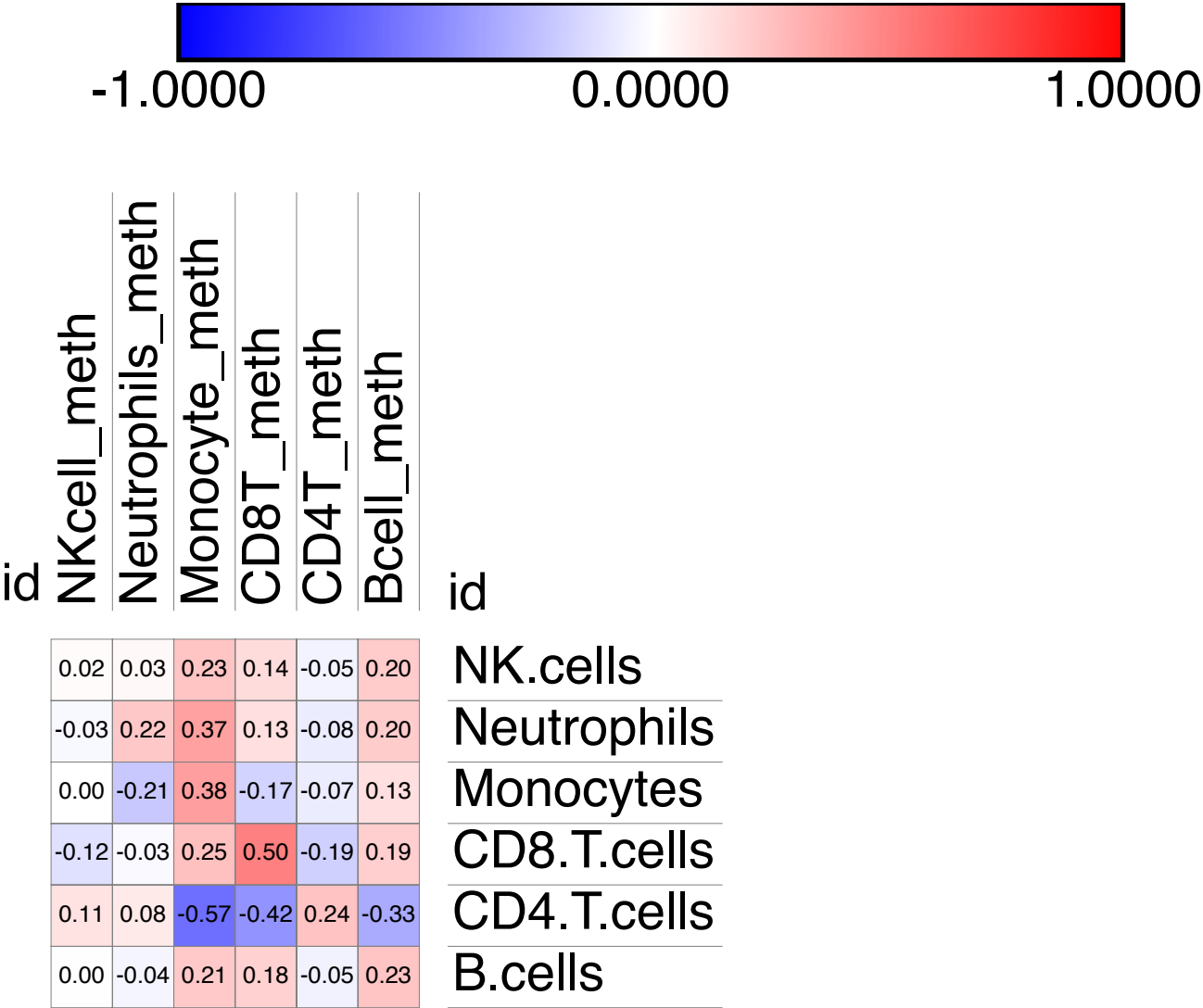

Supplement: Supplementary file 1 — Additional file 1: To validate our MethylCIBERSORT output we selected a data subset (N=394) with available gene expression data. CIBERSORTx method was used with LM6 signature matrix to get fraction of six major cell types. We found significant positive correlations between MethylCIBERSORT fraction and CIBERSORTx derived fraction of six major cell types. [file 40478_2021_1249_MOESM1_ESM.pdf]

## Supplementary Fig. 2

IDH wild type; N = 2072

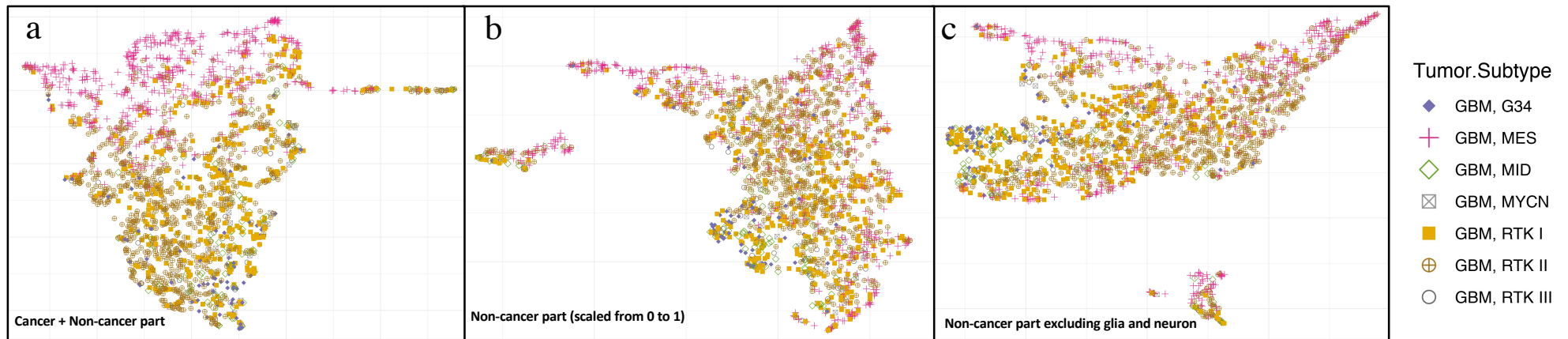

IDH mutant type; N = 1178

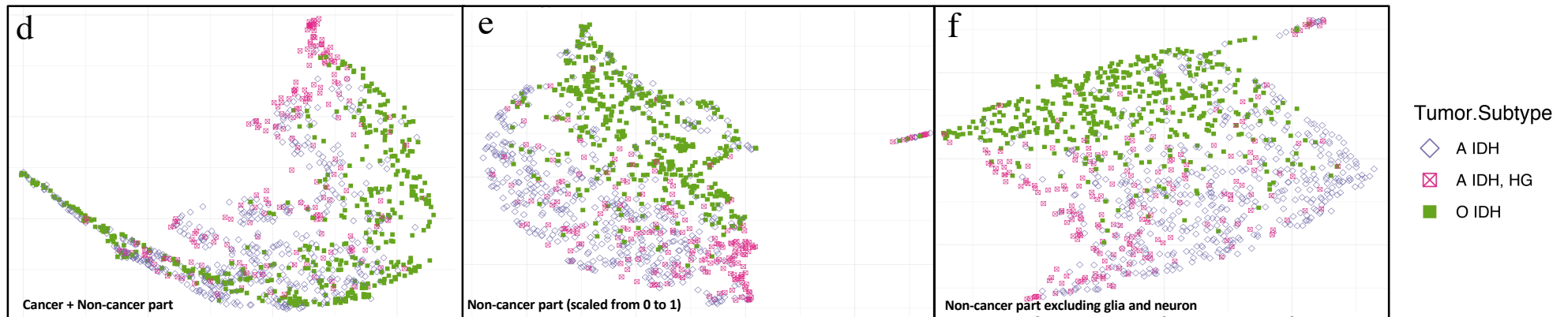

Low grade glioneuronal neoplasms; N= 802

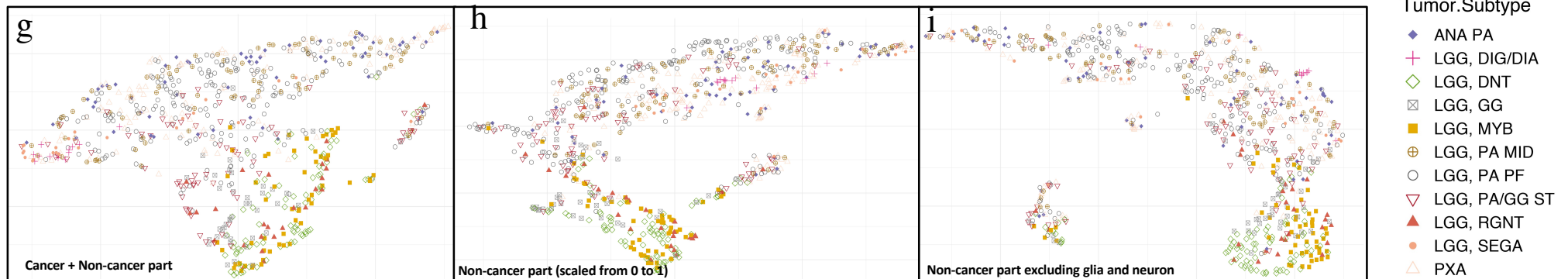

Supplement: Supplementary file 2 — Additional file 2: Assessment of clustering pattern in all three major cohorts IDH-wt (N=2072), IDH-mutant (N=1178) and Low-to-intermediate grade glioneuronal tumors (LIGGNT) (N=802) by including and excluding cancer part and scaling non-cancer part from 0 to 1. (a) UMAP clustering for IDH-wt cohort with all cell type (including cancer). (b) UMAP clustering for IDH-wt cohort with all normal cell type scaled 0 to 1 (non-cancer part). (c) UMAP clustering for IDH-wt cohort with all immune cells type scaled 0 to 1 (non-cancer part). (d) UMAP clustering for IDH-mutant cohort with all cell type(including cancer). (e) UMAP clustering for IDH-mutant cohort with all normal cell type scaled 0 to 1 (non-cancer part). (f) UMAP clustering for IDH-mutant cohort with all immune cells type scaled 0 to 1 (non-cancer part). (g) UMAP clustering for LIGGNT cohort with all cell type (including cancer). (h) UMAP clustering for LIGGNT cohort with all normal cell type scaled from 0 to 1 (non-cancer part). (i) UMAP clustering for LIGGNT cohort with all immune cells type scaled 0 to 1 (non-cancer part). [file 40478_2021_1249_MOESM2_ESM.pdf]

# Supplementary Fig. 3

a

|         | IDH-WT | IDH-MUT | O-IDH | A-IDH/HG | LIGGN  |
|---------|--------|---------|-------|----------|--------|
| kmeans  | 11 (5) | 8 (2)   | 6 (4) | 8 (5)    | 10 (2) |
| ward.D  | 8 (5)  | 10 (2)  | 8 (5) | 7 (2)    | 7 (2)  |
| ward.D2 | 8 (5)  | 8 (2)   | 5 (4) | 10 (3)   | 8 (2)  |

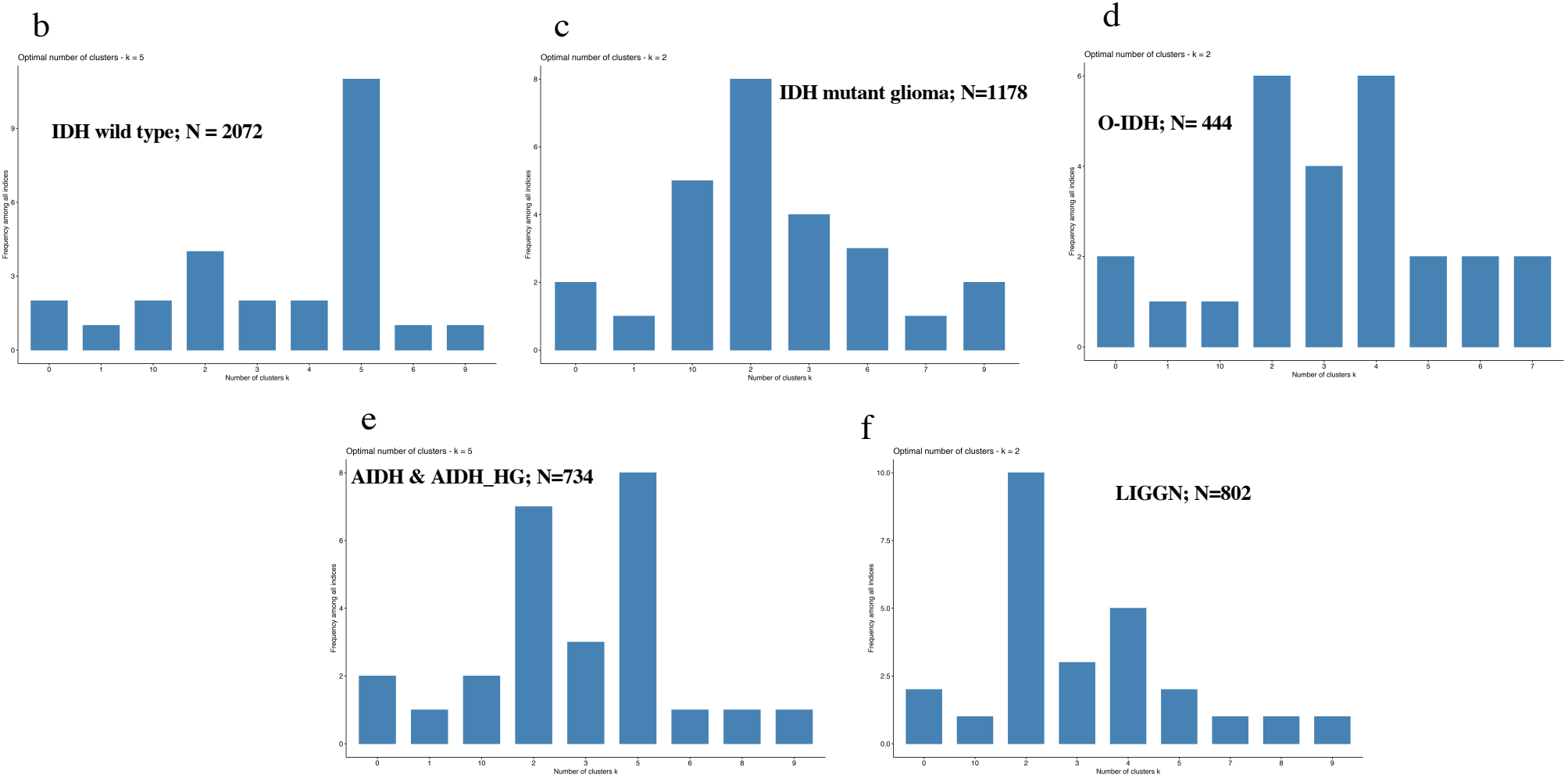

Supplement: Supplementary file 3 — Additional file 3: Cluster selection process was based on the majority rule, which is available in the NbClust package. We compared three different methods; kmeans, ward.D and ward.D2. (a) For each method we selected optimum number of clusters (given in brackets) proposed by maximum number of indices out of 30. We compared output of each method and found similar results. Finally, we selected kmeans clustering as a uniform approach to select optimum number of cluster (proposed by maximum number of indices out of 30) in each cohort. (b) In IDH wild type 11 indices proposed five cluster. (c) In IDH mutant type 8 indices proposed two cluster. (d) In O-IDH cohort, 6 indices proposed two and four clusters respectively. In this case we selected four cluster as ward.D2 method also suggested four clusters in O-IDH cohort. (e) In A-IDH/A-IDH-HG 8 indices proposed five clusters. (f) In LIGGNT 10 indices proposed two clusters. [file 40478_2021_1249_MOESM3_ESM.pdf]

Supplementary Fig. 4

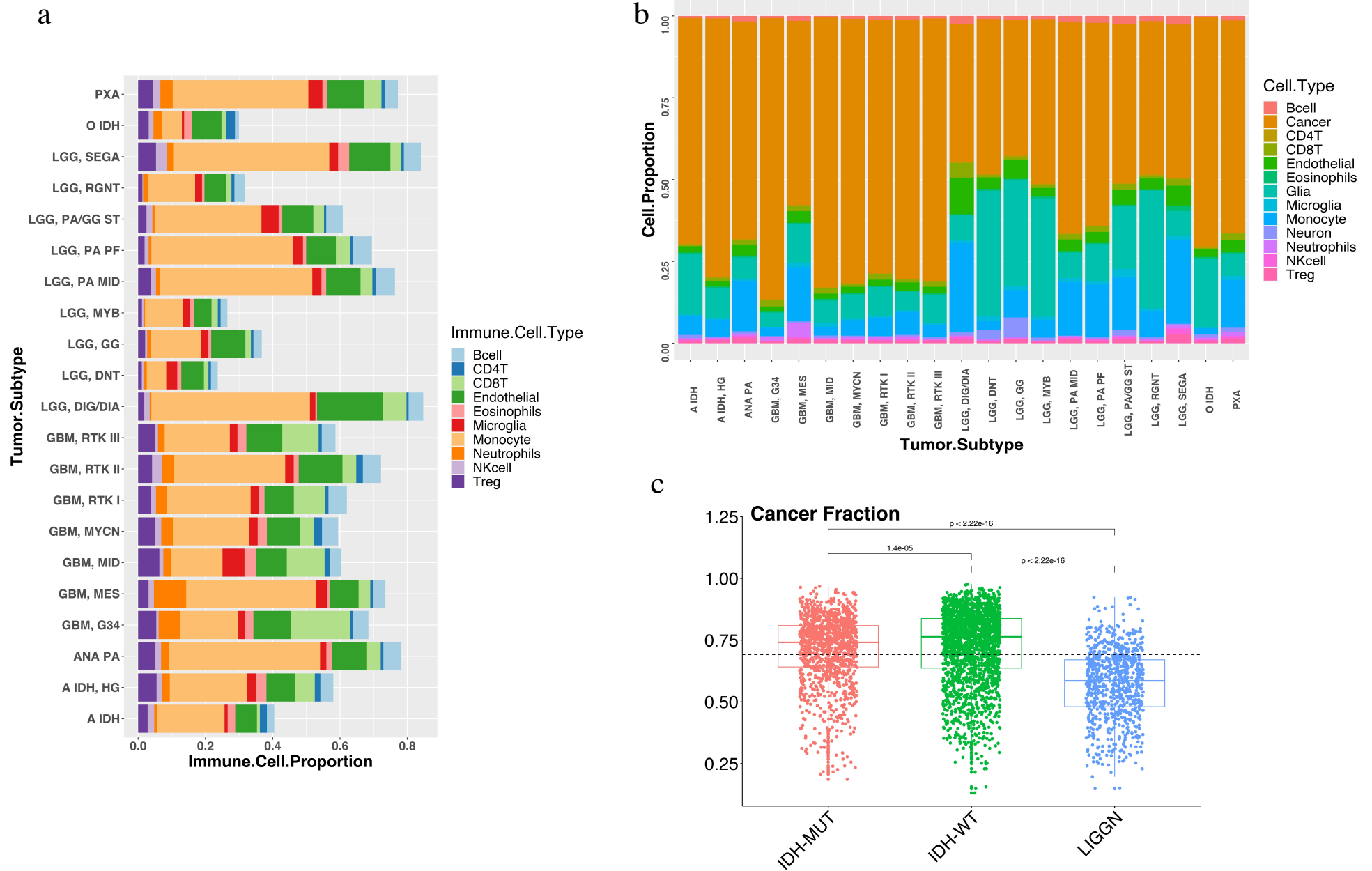

Supplement: Supplementary file 4 — Additional file 4: (a) Tumor subtype specific bar plot distribution of mean Immune cell proportion (Non-cancer part scaled 0 to 1, Glia and Neuron excluded). (b) Overall cell fractions of each cell type (Including Cancer, Glia and Neuron). (c) Cancer proportion shown by boxplots for each tumor subtype indicated significant differences. T-test and Wilcoxon test (p value shown) were used to calculate statistical significance. [file 40478_2021_1249_MOESM4_ESM.pdf]

**Supplementary Fig. 5**

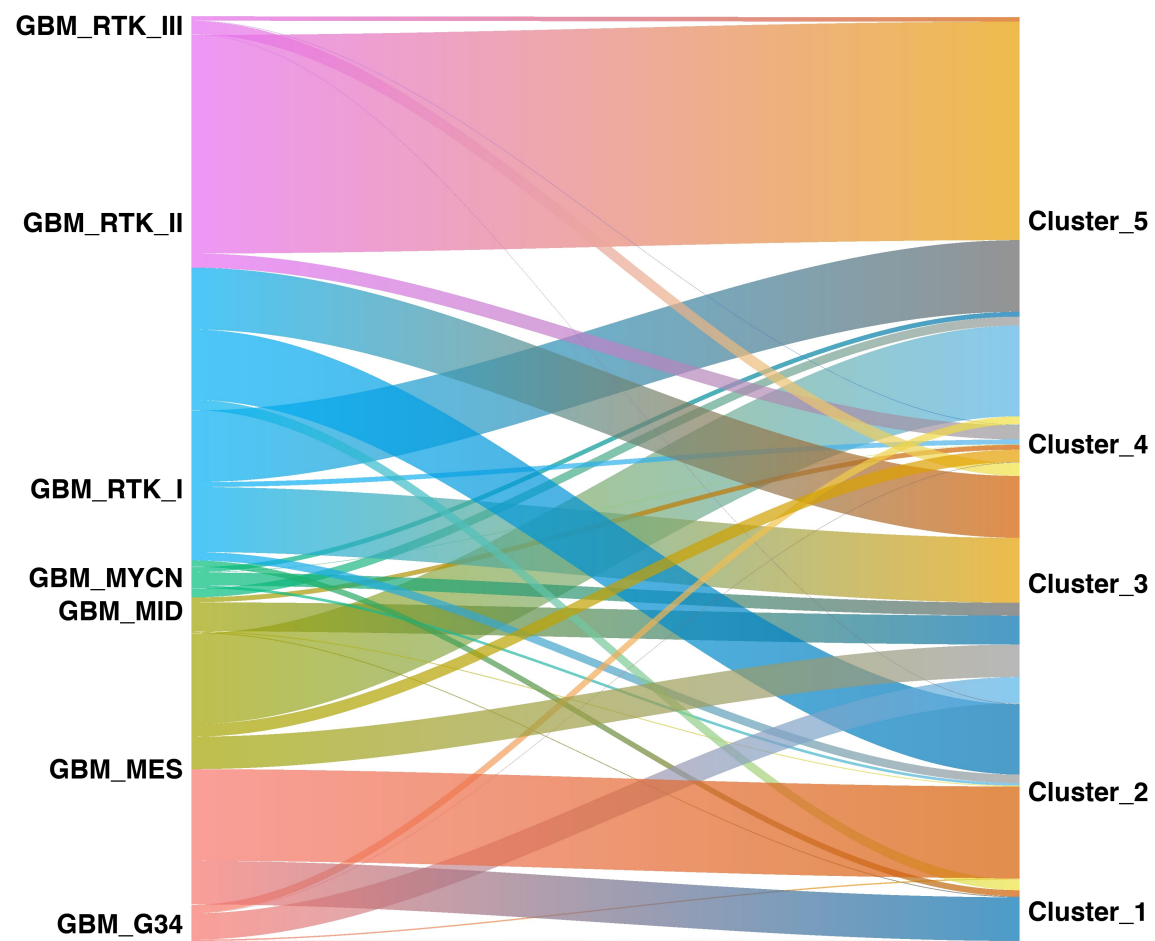

Supplement: Supplementary file 5 — Additional file 5: Sankey diagram-based associations between immune cells and tumor subtype in IDH-wt cohort (N=2072). Sankey plot showing proportions shared between each immune clusters and tumor subtype. [file 40478_2021_1249_MOESM5_ESM.pdf]

**Supplementary Fig. 6**

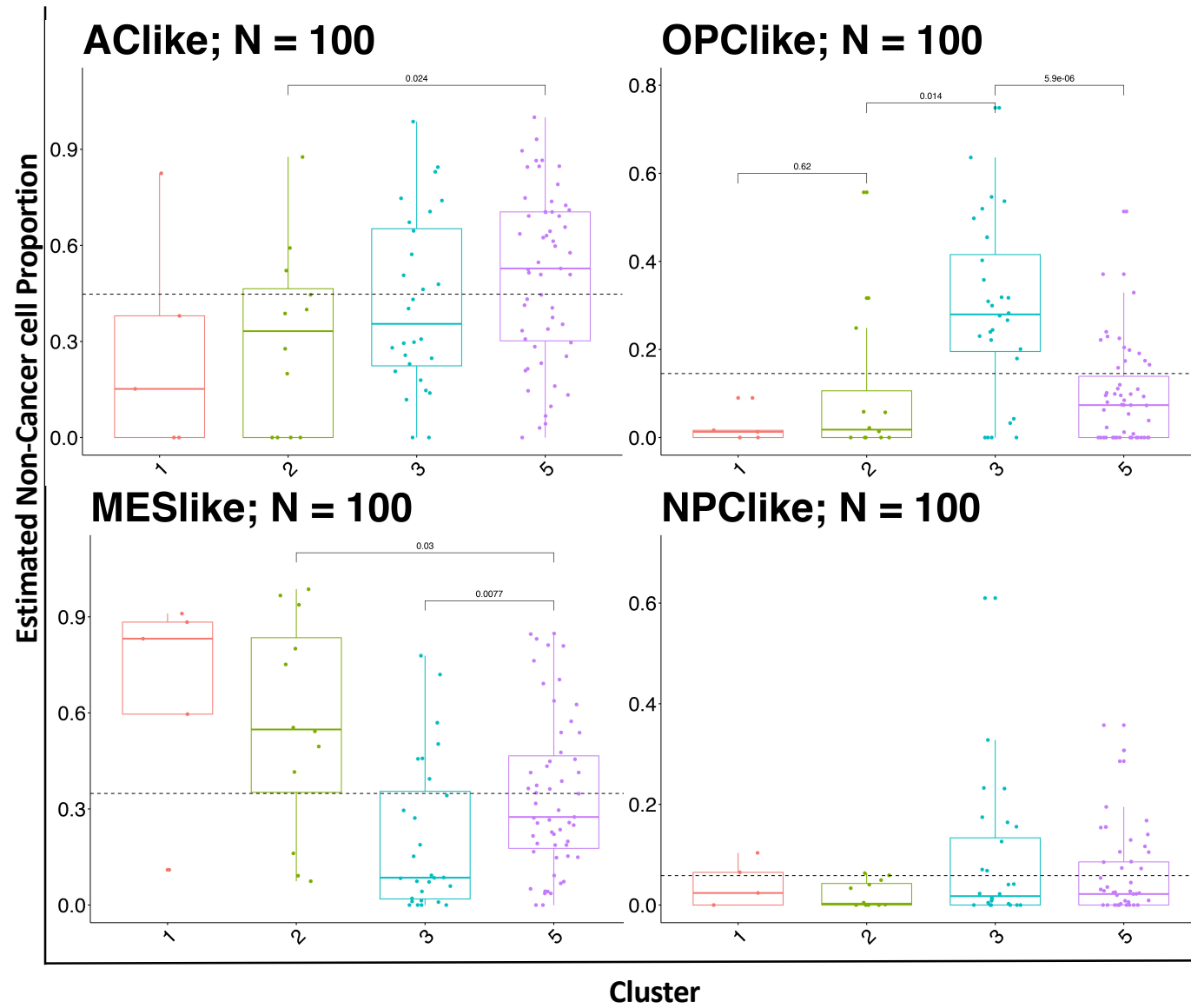

Supplement: Supplementary file 6 — Additional file 6: Analysis of the IDH-wt glioblastoma cohort (N=100) shows cluster specific distribution of AC-like, OPC-like, MES-like and NPC-like cellular states, respectively. Tumor cell states were derived from single cell data of IDH-wild type GBMs (PMID: 31327527). A signature matrix was derived and applied to TCGA samples for which gene expression data were available. Cell state estimations for each sample were performed using CIBERSORTx. T-test and Wilcoxon test (p-value shown) were used to calculate statistical significance. [file 40478_2021_1249_MOESM6_ESM.pdf]

# Supplementary Fig. 7

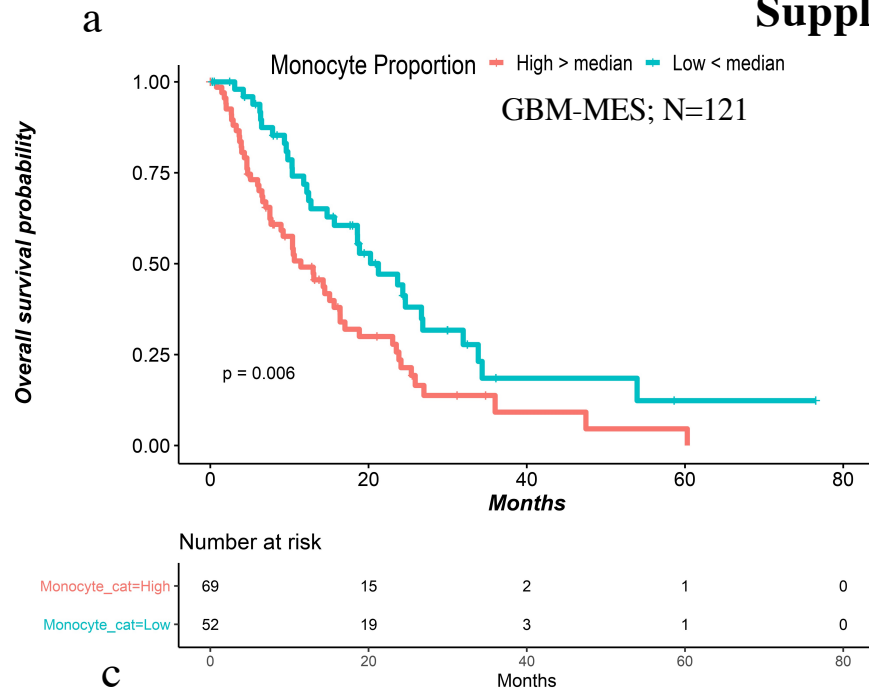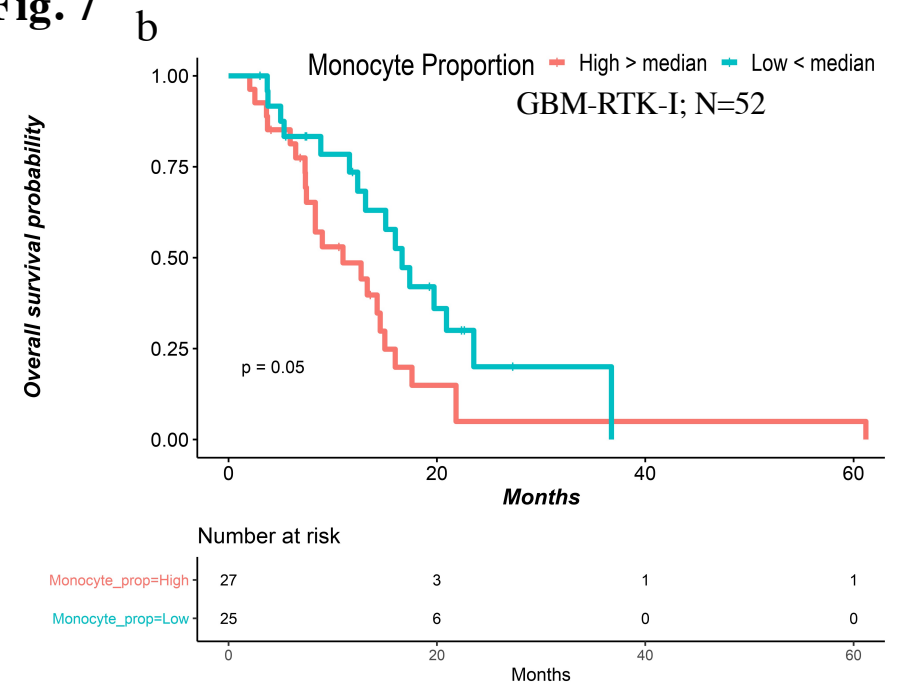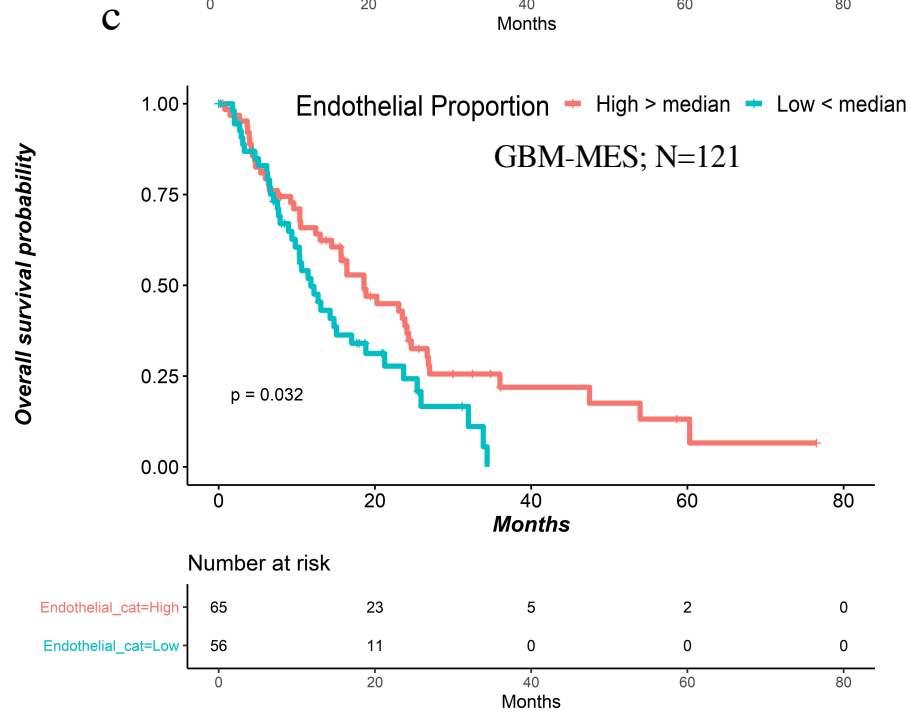

Supplement: Supplementary file 7 — Additional file 7: Kaplan-Meier plot of (a) GBM-MES for Monocyte proportion high and low groups (High>median, Low<median). (b) GBM-RTK-I dataset with high and low proportion of monocytes (High>median, Low<median). (c) Kaplan-Meier plot for Endothelial cell high and low groups in GBM-MES (High>median, Low<median). [file 40478_2021_1249_MOESM7_ESM.pdf]

**Supplementary Fig. 8**

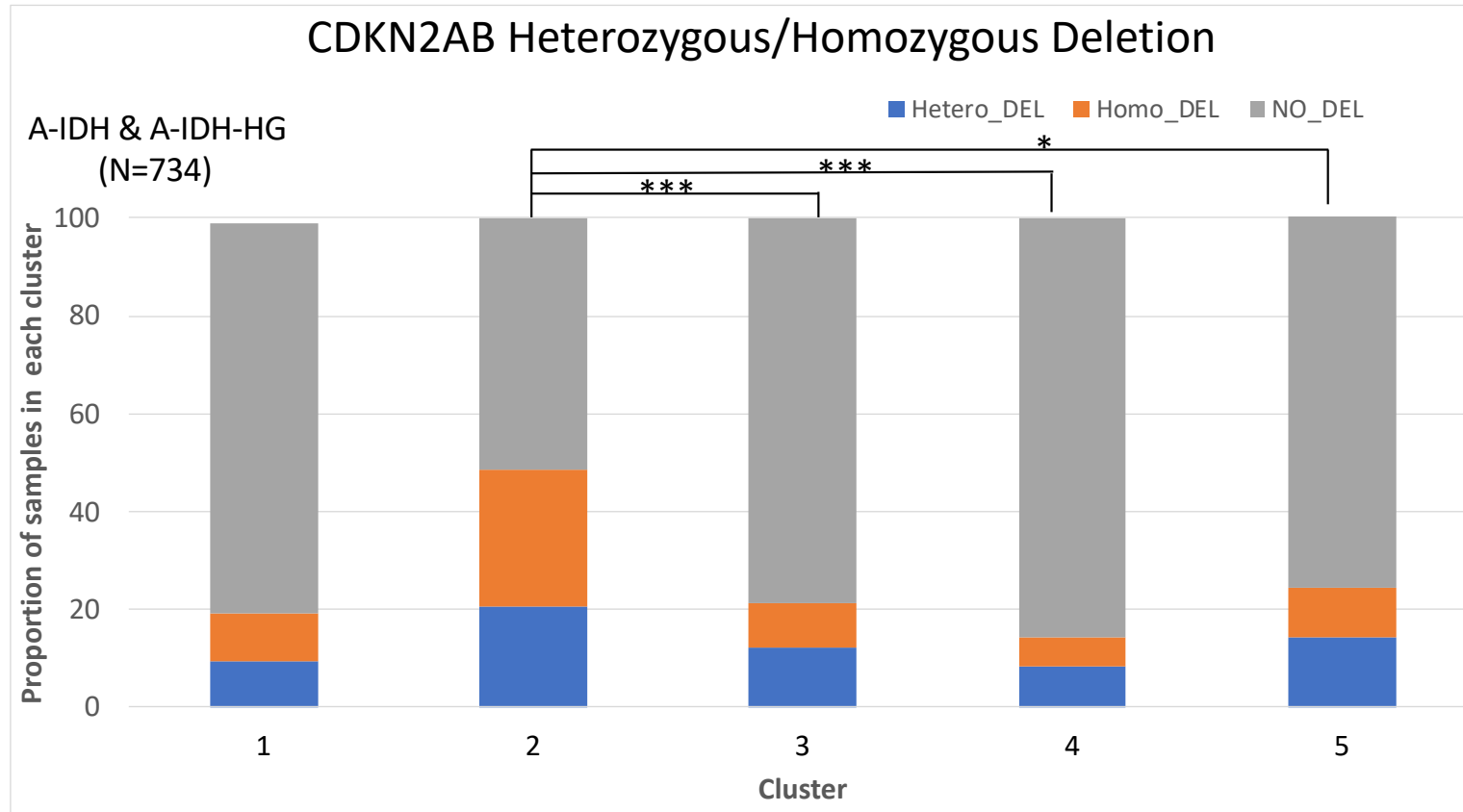

Supplement: Supplementary file 8 — Additional file 8: To investigate homozygous and heterozygous deletion of CDKN2AB as a separate event we selected a range of log2ratio and annotated samples as an event of homozygous deletion, heterozygous deletion, and no deletion. Significance was calculated by applying Fisher's exact test; p-value <(0.0001, 0.001, 0.01, 0.05, 1; symbols ="****", "***", "**", "*", "ns”). [file 40478_2021_1249_MOESM8_ESM.pdf]
